# Frequency and Clinical Aspects of Neurological and Psychiatric Symptoms in Patients with Non-Celiac Wheat Sensitivity

**DOI:** 10.3390/nu13061971

**Published:** 2021-06-08

**Authors:** Antonio Carroccio, Maurizio Soresi, Marta Chiavetta, Francesco La Blasca, Stella Compagnoni, Alessandra Giuliano, Francesca Fayer, Francesca Mandreucci, Daniele Castellucci, Aurelio Seidita, Andrea Affronti, Ada Maria Florena, Pasquale Mansueto

**Affiliations:** 1 Department of Health Promotion Sciences, Maternal and Infant Care, Internal Medicine and Medical Specialties (PROMISE), Unit of Internal Medicine, “V. Cervello Hospital”, University of Palermo, 90124 Palermo, Italy; martachiavetta@gmail.com (M.C.); stella.compagnoni.2011@gmail.com (S.C.); alegiuliano94@gmail.com (A.G.); francesca.mandreucci@gmail.com (F.M.); danicast94@hotmail.it (D.C.); 2 Department of Health Promotion Sciences, Maternal and Infant Care, Internal Medicine and Medical Specialties (PROMISE), Unit of Internal Medicine, University of Palermo, 90124 Palermo, Italy; maurizio.soresi@unipa.it (M.S.); francescolablasca@gmail.com (F.L.B.); francesca.fayer@gmail.com (F.F.); pasquale.mansueto@unipa.it (P.M.); 3 Department for the Treatment and Study of Abdominal Diseases and Abdominal Transplantation, IRCCS-ISMETT UPMC (University of Pittsburgh Medical Center), 90127 Palermo, Italy; xenis86@gmail.com; 4 Gastroenterology Unit, “V. Cervello Hospital”, 90124 Palermo, Italy; andrea.affronti@gmail.com; 5 Department of Health Promotion Sciences, Maternal and Infant Care, Internal Medicine and Medical Specialties (PROMISE), Pathology Unit, University of Palermo, 90124 Palermo, Italy; adamaria.florena@unipa.it

**Keywords:** non-celiac wheat sensitivity, irritable bowel syndrome, multiple food hypersensitivity, neuropsychiatric symptoms, HLA, duodenal lymphocytosis

## Abstract

Background: Non-Celiac Wheat Sensitivity (NCWS) is characterized by both intestinal and extra-intestinal symptoms. The study aims to investigate the frequency of neuropsychiatric manifestations in NCWS patients and identify their clinical and demographic characteristics. Methods: 278 clinical records of NCWS patients, diagnosed by a double-blind placebo-controlled wheat challenge between 2006 and 2020, were retrospectively revised. Fifty-two patients with Celiac Disease (CD) and 54 patients with Irritable Bowel Syndrome (IBS) served as controls. Results: 87% of the NCWS patients had an IBS-like clinical presentation. The NCWS group showed a longer duration of symptoms, a higher frequency of positive serum anti-nuclear antibodies than CD and IBS patients, and a higher frequency of DQ2/DQ8 haplotypes and duodenal mucosa lymphocytosis than IBS controls. In addition, 50% of NCWS patients showed neuropsychiatric manifestations, while lower percentages were observed in CD (25%) and IBS (28%) controls. Neuropsychiatric symptoms in NCWS were more frequently associated with the male sex, longer duration of symptoms, and IBS-diarrhea-like clinical presentation. Conclusions: Our data suggest that in patients with IBS-like symptoms and neuropsychiatric manifestations of unknown cause, it could be useful to investigate a correlation of these symptoms with wheat ingestion to identify NCWS patients with this ‘atypical’ manifestation.

## 1. Introduction

Abdominal pain, bloating, and altered bowel habits motivate up to 25% of all outpatient gastroenterological visits [1]. This clinical presentation is common to Celiac Disease (CD), Non-Celiac Wheat Sensitivity (NCWS), and Irritable Bowel Syndrome (IBS); therefore, differential diagnosis can be challenging. 

It is well known that CD symptoms are not limited to the gastrointestinal system but involve the whole body due to the malabsorption determined by the damage to the small intestine. Similarly, IBS and NCWS can have an extra-intestinal presentation. However, it remains unclear why some patients with CD or NCWS have only gastrointestinal symptoms, while others primarily or exclusively suffer from extraintestinal manifestations. 

In particular, neuropsychiatric disorders are often referred to and greatly impact the quality of life of the patients suffering from them. Ataxia and neuropathy are the disorders most frequently reported in untreated CD [2], with several studies and case reports describing an improvement in neurological symptoms after the start of a gluten-free diet (GFD) [3]. By contrast, depression, anxiety, and somatization disorders, together with headache and fatigue, are commonly referred by patients with IBS [4,5].

As concerns NCWS, some data report neurological manifestations similar to CD and equally responsive to GFD [2]. However, most of the studies in the literature do not differentiate between NCWS and CD. Hence, the true prevalence of neuropsychiatric symptoms in each disease is difficult to establish, and few data are available.

Our study aimed to analyze the frequency of neuropsychiatric symptoms in patients diagnosed with NCWS and any possible associations with their demographic characteristics and clinical features.

## 2. Materials and Methods

The clinical records of patients attending the outpatient centers of three Departments of Internal Medicine (at the “P. Giaccone” University Hospital and the “V. Cervello” Hospital in Palermo, Italy and at the “Giovanni Paolo II” Hospital of Sciacca, Italy) between January 2006 and December 2020, were retrospectively reviewed. These records are currently being uploaded into a computerized database. Among the clinical records already included in the database, there were 424 with a definitive diagnosis of NCWS made by double-blind -placebo-controlled (DBPC) wheat challenge. 

Inclusion criteria for NCWS patients were: (a) age >18 years; (b) NCWS diagnosis made by an elimination diet and subsequent DBPC wheat challenge; (c) complete clinical records; (d) follow-up duration longer than 9 months after the initial diagnosis and at least two outpatient visits during the follow-up period. Exclusion criteria of NCWS patients were no compliance with the (a)–(d) inclusion criteria and pregnancy. A total of 278 patients were considered eligible for our study (see Supplementary Figure S1).

Two control groups were recruited in the same centers. The first was composed of 52 CD patients and the second of 54 IBS patients. Both were randomly chosen by a computer-generated method from subjects diagnosed during the same period (2006–2020), and age (±2 years) and sex-matched (±5%) with the NCWS patients.

The following criteria were adopted to diagnose NCWS. Firstly, organic gastrointestinal diseases—in particular CD and WA—were excluded. As a result, all the patients had negative serum anti-tissue transglutaminase (anti-TTG) and anti-endomysial (EMA) IgA and IgG antibodies, absence of villous atrophy in duodenal samples (collected in all subjects carrying the DQ2 or DQ8 HLA haplotypes and in others when clinically appropriate), and IgE-mediated immune-allergy tests negative to wheat (skin prick tests and/or serum specific IgE detection). Inflammatory bowel diseases were also excluded. For details, see Supplementary Materials. After other organic diseases had been excluded, NCWS was diagnosed using the following criteria: the resolution of symptoms after a standard elimination diet excluding wheat, cow’s milk, or other foods causing self-reported symptoms, and their reappearance on DBPC wheat challenge, performed as previously described [6,7] (for details, see Supplementary Materials). Other “open” food challenges were also performed.

As regards the control groups, the CD was diagnosed according to the current guidelines [8], and IBS was diagnosed according to the Rome IV criteria [9]. The IBS controls included in this study had been receiving the same elimination diet as the NCWS patients but had not shown any clinical improvement. 

As concerns neurological disorders, we only considered self-reported neuropsychiatric symptoms that disappeared on a wheat-free diet and reappeared after wheat ingestion on the DBPC challenge. Our evaluation did not include overt neurological and psychiatric diseases diagnosed by specific tests (imaging, electroencephalogram, electromyography, electroneurography), or symptoms which could be related to any other risk factors known at the time of our study (i.e., diabetes, vitamin deficiencies, exposure to neurotoxic agents) and not related to wheat ingestion. 

For each patient, the following clinical features were analyzed: (1) age at diagnosis, (2) duration of the symptoms (months), (3) body mass index (BMI), (4) presence and kind of IBS-like presentation, (5) presence of dyspepsia, (6) body weight loss (defined as a 10% reduction in body weight in six months or less), (7) anemia (defined as hemoglobin <12 g/dL for women and <13 g/dL for men), (8) presence of concomitant autoimmune diseases, (9) serum anti-nuclear antibody (ANA) positivity (at titer ≥1:80), (10) presence of cow’s milk sensitivity, evaluated by DBPC challenge, (11) multiple food hypersensitivity (MFH), other than to wheat and cow’s milk, evaluated by open challenges, (12) allergic nickel dermatitis, evaluated by patch tests, (13) concurrent atopy (defined as a history of allergic rhinoconjunctivitis and/or asthma and/or atopic dermatitis), and (14) presence of the HLA DQ2/DQ8 haplotypes. Furthermore, duodenal histology at baseline was evaluated in patients with HLA DQ2/DQ8 or other patients when considered clinically appropriate. Intraepithelial lymphocyte (IEL) counts >25/100 epithelial cells were classified as Marsh 1 lesions [10]. 

The study was approved by the Ethics Committee of the University of Palermo, Italy. Due to the retrospective design of the study, patients were not consulted. However, we received comments from four patients included in the study during the review and revised our manuscript accordingly. The study was registered at clinicaltrials.gov (registration number NCT04769180).

### Statistical Analysis

Data were expressed as mean ± standard deviation (SD) when distribution was Gaussian, and differences were calculated using Student’s *t*-test. Otherwise, data were expressed as median and range and analyzed with the Mann-Whitney U test. Fisher’s exact test or the Chi-square test were used where appropriate.

## 3. Results

The demographic and clinical features of the study patients are shown in Table 1.

Two hundred and seventy-eight patients were diagnosed with NCWS and 87% were female. In addition, the median duration of symptoms from onset to diagnosis was much longer in the NCWS group (60 months) than in the CD (12 months) and IBS (30 months) groups (NCWS vs. CD *p* = 0.0001; NCWS vs. IBS *p* = 0.04).

Eighty-seven percent of the NCWS study patients showed IBS-like symptoms, a frequency significantly higher than in CD controls (*p* = 0.03). Among the NCWS patients with IBS-like symptoms, 143 (51.4%) had IBS-diarrhea, 36 (12.9%) IBS-constipation, and 63 (22.7%) IBS with alternate bowel movements.

CD patients reported weight loss significantly more often than NCWS and IBS (CD 44% vs. NCWS 25% vs. IBS 18%; *p* = 0.01 for both). Interestingly, NCWS patients showed a significantly higher frequency of weight loss and anemia than IBS controls (*p* = 0.01 and 0.005, respectively). This finding was confirmed by the BMI values (CD 21.6 ± 5.1 vs. NCWS 24.1 ± 5.2 vs. IBS 26.5 ± 6.2; CD vs. IBS *p* = 0.02).

Meanwhile, fifty-six NCWS patients showed one or more concurrent autoimmune diseases. Hashimoto’s thyroiditis was the most frequent, being present in 42 NCWS patients. A higher percentage of positive ANA was also found in NCWS patients than in CD and IBS (NCWS 47% vs. CD 30%, *p* = 0.03; NCWS vs. IBS 5%, *p* = 0.0001). 

Cow’s milk sensitivity was significantly more frequent in the NCWS group (64%) than in CD (23%) and IBS (22%) controls (*p* = 0.0001 for both). Similarly, intolerances towards foods other than wheat and cow’s milk were more frequent in NCWS than in the control groups (NCWS 36% vs. CD 4%, *p* = 0.0001; NCWS vs. IBS 11%, *p* = 0.0005). 

HLA DQ2/DQ8 haplotypes were significantly more frequent in NCWS than in the IBS controls (55% vs. 30%, *p* = 0.001). Similarly, duodenal mucosa intraepithelial lymphocytosis (Marsh 1 lesion) was more frequent in NCWS than in the IBS controls (*p* = 0.02).

Exactly half of the patients with NCWS reported neuropsychiatric symptoms (139/278), and the frequency of these symptoms in the NCWS group was greater than in the CD and IBS controls (NCWS 50% vs. CD 25%, *p* = 0.002, NCWS vs. IBS 28%, *p* = 0.005). The demographic and clinical features of the NCWS patients with or without neuropsychiatric symptoms are shown in Table 2. 

**Figure 1:**
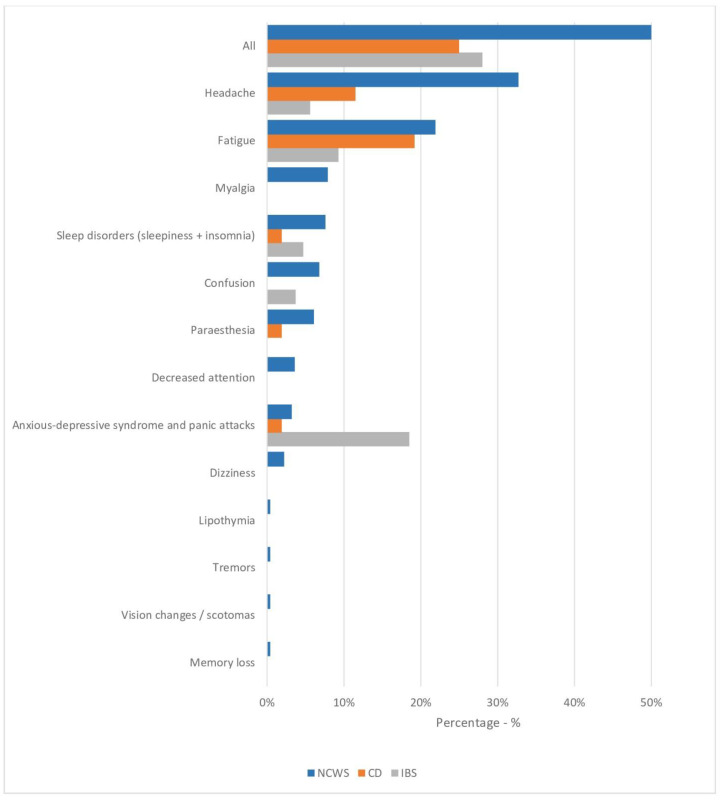
Frequency of each neurologic and psychiatric symptom in NCWS patients (*n* = 278) and in CD (*n* = 52) and IBS (*n* = 54) controls included in the study. Data are expressed as percentages.

The NCWS patients with neuropsychiatric symptoms were diagnosed later (approximately one year later, *p* = 0.05), and the proportion of men was higher (*p* = 0.02) than in the NCWS group without neuropsychiatric symptoms. Moreover, although the frequency of the IBS-like clinical presentation was similar in the two groups, IBS-diarrhea was observed more frequently among NCWS patients with neurological symptoms (59% vs. 44%, *p* = 0.02). In addition, there was a higher frequency of body weight loss, anemia, and ANA positivity in NCWS patients with neuropsychiatric symptoms than those without, but no statistically significant differences were observed. Finally, neither HLA DQ2/DQ8 positivity nor duodenal inflammation (Marsh 1 lesion) was significantly different in NCWS patients with or without neuropsychiatric symptoms. The frequency of each neuropsychiatric symptom in the three groups is shown in Figure 1.

Headache was the most frequently reported neuropsychiatric symptom in the NCWS group, with a significantly higher percentage than in the two control groups (*p* = 0.005 for both). Fatigue was reported in similar percentages in the NCWS and CD patients, which were higher for both these groups than in IBS controls (NCWS 21.9% vs. CD 19.2% vs. IBS 9.3%, NCWS vs. IBS *p* = 0.03). Myalgia was recorded in 7.9% of NCWS patients with a significantly higher frequency than in the CD and IBS control groups (*p* = 0.03 for both). On the contrary, psychiatric disorders, such as anxiety, depression, and panic attacks, were more frequent in the IBS group (NCWS 3.2% vs. CD 1.9% vs. IBS 18.5%, NCWS vs. IBS *p* = 0.03, CD vs. IBS *p* = 0.01).

## 4. Discussion

NCWS is an emerging clinical condition that has increasingly attracted the interest of researchers in the last few years. In our study, we retrospectively included 278 patients diagnosed with NCWS by the DBPC wheat challenge. The clinical characteristic of these subjects recalled some of those reported in previous studies. First of all, this condition seems to be much more frequent, or more often self-reported, among women in the fourth decade of life [11,12,13,14,15], and the same trend of prevalence emerged from our study (females 87%, mean age at diagnosis 37.9 ± 12.4 years). Another interesting issue emerging from our study concerns the delay of NCWS diagnosis. The NCWS group showed a statistically significant delay in diagnosis compared to CD (60 vs. 12 months, *p* = 0.0001) and IBS (60 vs. 30 months, *p* = 0.04). This delay may be due to the lack of diagnostic markers for NCWS and that the DBPC challenge, which is the current gold standard for the diagnosis, is not easily performed, except in tertiary centers specializing in gluten-related diseases. 

As already reported in the literature, at onset, the symptoms of NCWS patients are similar to those of IBS [16]. In our study group, 87% of the patients showed the clinical criteria for an IBS diagnosis. The frequency of autoimmune disease in the NCWS group (20%) was in agreement with the frequency reported in previous studies of our group [7,17], and a higher percentage of ANA positivity was found in the NCWS group than the controls (NCWS 47% vs. CD 30% vs. IBS 5%). MFH frequency was significantly higher in the NCWS patients than in CD and IBS. This is in keeping with our hypothesis that NCWS subjects might be suffering from a non-IgE-mediated food allergy [18]. The NCWS patients had a higher frequency of HLA DQ2/DQ8 haplotypes than the IBS controls (55% vs. 30%) and this percentage is close to that reported in the literature [2,19]. A further point that suggested an inflammatory condition contributing to NCWS pathogenesis was the duodenal mucosa lymphocytosis observed in 52% of the NCWS patients included in the present study, a percentage significantly higher than in the IBS controls.

Due to the prevalent IBS-like clinical presentation in NCWS patients, most previous studies have focused on improving a wheat-free diet on the intestinal symptoms. However, although extra-intestinal symptoms have also been reported in NCWS, few studies have reported their frequency. In addition, no previous studies, to our knowledge, have evaluated the clinical characteristics of NCWS patients with neurological manifestations referred to Internal Medicine or Gastroenterology Units. 

We found that 139 (50%) of the NCWS patients showed at least one neuropsychiatric symptom which disappeared on the wheat-free diet and reappeared on the DBPC wheat challenge. Neuropsychiatric symptoms were found to be more frequent in NCWS patients than in CD or IBS patients (respectively NCWS 50% vs. CD 25%, *p* = 0.002; NCWS vs. IBS 28% *p* = 0.005). Since the duration of symptoms from onset to diagnosis was longer in the NCWS patients than in CD controls, this might suggest that the duration of wheat exposure could determine a greater risk for developing neuropsychiatric symptoms. However, to the best of our knowledge, there are no other available data about this issue. In fact, in the literature, gluten-related neurological disorders are better described in patients with CD than with NCWS [20]. Nevertheless, another study reported that patients with CD are more likely to develop neurological symptoms earlier than NCWS patients [2]. 

Among the NCWS patients, there was a higher prevalence of the male sex in the group with neurological symptoms (18% vs. 7.2%, *p* = 0.02). Of the total 35 male patients with NCWS included in the study, 25 complained of neurological symptoms. This finding agrees with the higher prevalence of male sex observed among the patients analyzed in a recent systematic review, which showed that the majority of gluten-related neurological disorders, proven by histological findings on nervous tissue biopsy or autopsy, affected men [21]. 

It is also interesting to note that in the NCWS group, the patients with neuropsychiatric symptoms received a later diagnosis (approximately one year later) than those without neurological manifestations. This finding is in keeping with the literature, which reports that CD patients presenting with neurological manifestations are likely to be diagnosed significantly later than those presenting with gastrointestinal symptoms [2]. 

Very little is known about the pathogenesis of gluten/wheat-related neurological manifestations. Still, several studies have suggested a major role for the emerging concept of a “microbiota-gut-brain axis”: according to this hypothesis, there is a close connection between the gut microbiota and the central nervous system, with the former regulating the central nervous system’s functioning via neural, immune, and endocrine pathways. Moreover, the gut microbiota might reduce some neurotoxic intermediates or immunogenic wheat proteins and delay the onset or reduce the severity of neurodegenerative disorders [22,23,24]. 

One of the etiopathogenetic hypotheses for gluten-related disorders is known as “the leaky gut hypothesis.” Gluten is identified as the element that triggers an innate autoimmune response, which feeds an inflammatory process affecting the intestinal mucosa, reducing the number of tight junction proteins and increased permeability of the intestinal barrier. This results in the passage of immunogenic molecules into the peripheral circulation. Thus, both the diffusion of gluten-derived peptides in the blood and the immunologic activation driven by other wheat proteins could justify the development of the extra-intestinal manifestations of these diseases, which then take on the characteristics of real syndromes [25,26,27]. 

However, in our study, we observed that the NCWS patients with neurological symptoms showed a higher prevalence of IBS-diarrhea symptoms than those without neurological symptoms. This could suggest that symptoms may be due to the malabsorption of trace elements important for proper central nervous system functioning. 

As regards the specific symptoms recorded in our study groups, the headache was the most common symptom among NCWS patients (32.7% vs. CD 11.5%, *p* = 0.02; vs. IBS 5.6%, *p* = 0.0001), followed by fatigue, which was significantly more frequent in NCWS than in the IBS group (*p* = 0.03). In addition, myalgia was recorded in 7.9% of the NCWS patients, with a significantly higher frequency than in the CD and IBS control groups. On the contrary, anxiety and depression were more common in IBS than in NCWS patients.

Although our study provides relevant data on the frequency and clinical aspects of the neuropsychiatric manifestations due to wheat ingestion in NCWS patients, its limits must be underlined. First, it is a retrospective study, and the results need to be confirmed in a prospective design. Second, no neuroimaging studies were performed, and we cannot affirm that the neuropsychiatric symptoms were linked to real neuronal damage. Third, we have no data about the patients’ intestinal microbiota, changes in which, driven by the wheat-containing or the wheat-free diet, could have important effects on the gut-brain axis. Fourth, our data were not collected in neurology clinics. Almost all our patients had gastrointestinal symptoms as the most relevant clinical presentation. Thus, our estimation of the frequency and severity of the neuropsychiatric manifestations probably applied to patients with mild existing neuropsychiatric symptoms.

The strong point of the study was that it included a large number of NCWS patients diagnosed by DBPC wheat challenge and well defined by additional clinical and laboratory data. 

## 5. Conclusions

In conclusion, we found that 50% of the NCWS patients with mainly gastrointestinal symptoms also suffered from neuropsychiatric manifestations due to wheat ingestion. Male sex, a longer duration of symptoms, and IBS-diarrhea were associated with neuropsychiatric symptoms. Among them, headache, fatigue, and myalgia were the most common. Our data suggest that in patients with IBS-like symptoms and neuropsychiatric manifestations of unknown cause or poorly responsive to drugs, it could be useful to investigate a possible correlation of these symptoms with wheat ingestion to identify patients with CD or NCWS with this ‘atypical’ manifestation. Further studies will be needed to clarify the pathogenesis of gluten-related neuropsychiatric manifestations and their correlation with the clinical features of NCWS patients to enable an earlier diagnosis.

## Figures and Tables

**Table 1:** Demographic and clinical features of the patients with NCWS, CD, and IBS.

	NCWS (*n* = 278) (%)	CD (*n* = 52) (%)	IBS (*n* = 54) (%)	*p*
Female Sex	243 (87%)	46 (88%)	47 (88%)	NS
Age at diagnosis (mean ± SD; years)	37.9 ± 12.4	38.9 ± 14.8	40.5 ± 14.3	NS
Duration of the symptoms (median and range; months)	60 (0–612)	12 (1–732)	30 (6–360)	NCWS vs. CD 0.0001
				NCWS vs. IBS 0.04
Body Mass Index (mean ± SD)	24.1 ± 5.2	21.6 ± 5.1	26.5 ± 6.2	CD vs. IBS 0.02
Presence of IBS-like symptoms	242 (87%)	38 (73%)	54 (100%)	NCWS vs. CD 0.03
				NCWS vs. IBS 0.02
				CD vs. IBS 0.0002
Dyspepsia	155 (56%)	38 (73%)	33 (61%)	NS
Body weight loss	70 (25%)	23 (44%)	10 (18%)	NCWS vs. CD 0.01
				NCWS vs. IBS 0.01
				CD vs. IBS 0.01
Anemia	84 (30%)	31 (59%)	6 (11%)	NCWS vs. CD 0.0001
				CD vs. IBS 0.005
Autoimmune diseases	56 (20%)	11 (21%)	7 (13%)	NS
Hashimoto’s thyroiditis	42 (15%)	6 (12%)	5 (9%)	NS
Other AD	23 (8%)	5 (10%)	3 (6%)	NS
Positive serum ANA	130 (47%)	16 (30%)	3 (5%)	NCWS vs. CD 0.03
				NCWS vs. IBS 0.0001
				CD vs. IBS 0.0001
Cow’s milk sensitivity	177 (64%)	12 (23%)	12 (22%)	NCWS vs. CD 0.0001
				NCWS vs. IBS 0.0001
Multiple food hypersensitivity(other than cow’s milk)	101 (36%)	2 (4%)	6 (11%)	NCWS vs. CD 0.0001
				NCWS vs. IBS 0.0005
Allergic nickel dermatitis	45 (16%)	5 (10%)	4 (7%)	NS
Atopic diseases	93 (33%)	13 (25%)	16 (29%)	NS
HLA DQ2/DQ8 haplotypes	154 (55%)	52 (100%)	16 (30%)	NCWS vs. CD 0.0001
				NCWS vs. IBS 0.001
				CD vs. IBS 0.0001
Marsh				
0	83 (48%)	0 (0%)	7 (100%)	
1	88 (52%)	10 (18%)	0 (0%)	Frequency of Marsh 1 lesion:
				NCWS vs. IBS 0.02
2	0 (0%)	0 (0%)	0 (0%)	
3	0 (0%)	42 (82%)	0 (0%)	Frequency of Marsh 3 lesion:
				NCWS vs. CD 0.0001
				CD vs. IBS 0.0001

Abbreviations: NCWS = Non-Celiac Wheat Sensitivity; CD = Celiac Disease; IBS = Irritable Bowel Syndrome; SD = Standard Deviation; AD: Autoimmune Diseases; ANA = Anti-Nuclear Antibodies; HLA = Human Leukocyte Antigen; NS = Non-Significant.

**Table 2:** Demographic and clinical features of NCWS patients with neurological symptoms compared to NCWS without neurological symptoms.

	NCWS Patients without Neuropsychiatric Symptoms (*n* = 139) (%)	NCWS Patients with Neuropsychiatric Symptoms (*n* = 139) (%)	*p*
Male sex	10 (7.2%)	25 (18%)	0.02
Age at diagnosis (mean ± SD; years)	37.7 ± 13.1	37.9 ± 11.7	NS
Duration of symptoms (median and range; months)	60 (2–612)	72 (0–564)	0.05
Body Mass Index (mean ± SD)	23.8 ± 4.8	24.3 ± 5.4	NS
Presence of IBS-like symptoms	119 (86%)	123 (89%)	NS
Dyspepsia	84 (60%)	71 (51%)	NS
Body weight loss	28 (20%)	42 (30%)	NS
Anemia	49 (25%)	35 (35%)	NS
Autoimmune diseases	28 (20%)	28 (20%)	NS
Hashimoto’s thyroiditis	18 (13%)	24 (17%)	NS
Other AD	13 (9%)	10 (7%)	NS
Positive serum ANA	60 (43%)	70 (50%)	NS
Cow’s milk sensitivity	85 (61%)	92 (66%)	NS
Multiple food hypersensitivity (other than cow’s milk)	45 (32%)	56 (40%)	NS
Allergic nickel dermatitis	20 (14%)	25 (18%)	NS
Atopic diseases	47 (34%)	51 (37%)	NS
HLA DQ2/DQ8 haplotypes	79 (57%)	75 (54%)	NS
Marsh			
0	42/87 (48%)	41/84(49%)	NS
1	45/87 (52%)	43/84 (51%)	NS

Abbreviations: NCWS = Non-Celiac Wheat Sensitivity; SD = Standard Deviation; IBS = Irritable Bowel Syndrome; AD: Autoimmune Diseases; ANA = Anti-Nuclear Antibodies; HLA = Human Leukocyte Antigen; NS = Non-Significant.

## Data Availability

The data presented in this study are available on request from the corresponding author. The data are not publicly available due to privacy.

## Supplementary Materials

The following are available online at ’https://www.mdpi.com/article/10.3390/nu13061971/s1; Figure S1: Flow Chart of the Study. Supplementary Material: Exclusion of CD and IBD and diagnosis of NCWS.
