# Supplementary material for: Frequency and Clinical Aspects of Neurological and Psychiatric Symptoms in Patients with Non-Celiac Wheat Sensitivity
Source: Nutrients. 2021 Jun 8;13(6):1971. doi: 10.3390/nu13061971 (PMC8227001; doi:10.3390/nu13061971)
Supplement: Supplementary file 1 [file nutrients-13-01971-s001.zip › nutrients-1202602-supplementary.pdf]

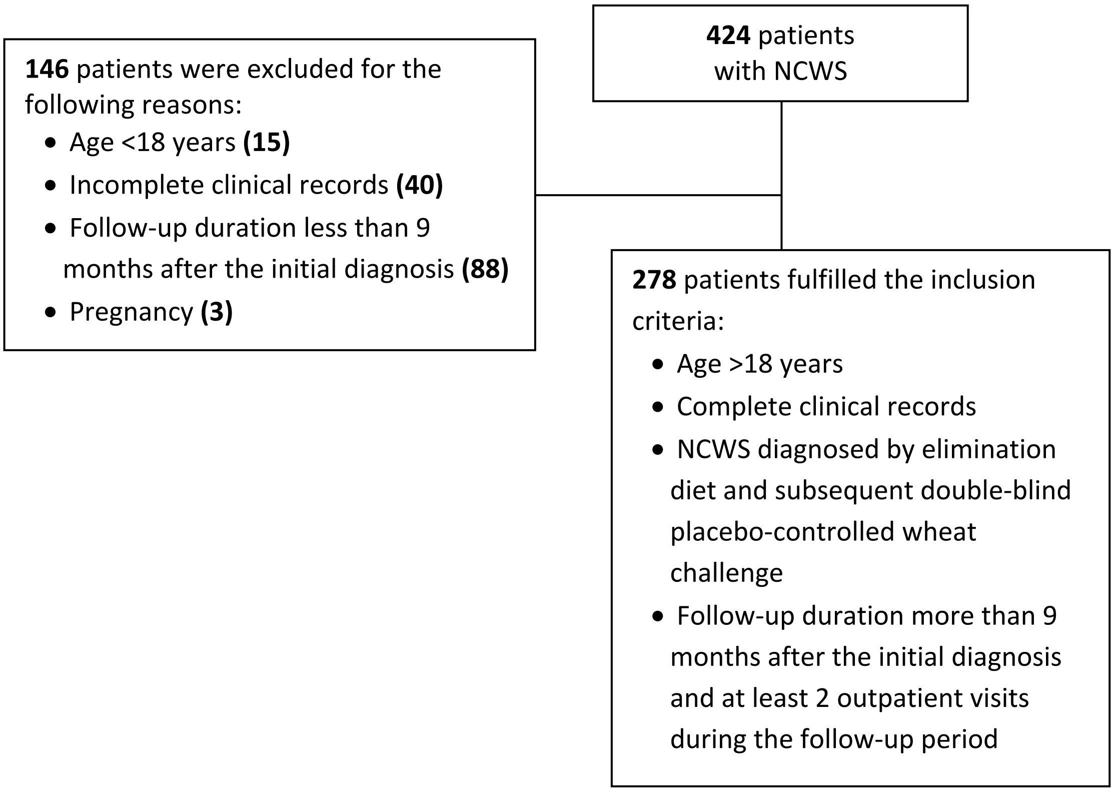

Figure S1: Flow Chart of the Study: Number of patients excluded from and included in the study.

Exclusion of Celiac Disease (CD) diagnosis

Before diagnosis, patients were instructed to eat foods containing wheat, consuming at least five slices of wheat bread per day (about 8 grams of gluten) for four weeks. At the end of this period, all patients underwent assays for serum anti-transglutaminase (anti-tTG) IgA and IgG, performed using commercial kits (Eu-tTG IgA, and anti-gliadin IgA and IgG, Eurospital Pharma, Trieste, Italy). Patients were also typed for HLA-DQ phenotypes by polymerase chain reaction, using sequence-specific primers with a rapid detection method (DQ-CD Typing Plus kit by BioDiaGene, Palermo, Italy). Patients positive for the DQ2 and/or the DQ8 haplotypes also underwent duodenal mucosa biopsy, regardless of the results of the CD- specific antibody assay.

CD diagnosis was excluded when: A) DQ2 and/or DQ8 haplotypes were absent or B) anti-tTG IgA and IgG were negative and duodenal histology showed a normal villus/crypt ratio (≥ 3). On the contrary, CD diagnosis was not excluded if patients were positive at anti-endomysium (EMA) assay of the culture medium of the duodenal biopsies, even if the villus/crypt ratio in the duodenal mucosa was normal. Consequently, these patients were not included in the Non-Celiac Wheat Sensitivity (NCWS) group.

Exclusion of Inflammatory Bowel Disease (IBD) diagnosis

IBD diagnosis was excluded when serum C reactive protein, erythrocyte sedimentation rate and white blood cell count were normal in repeated examinations, performed when the patients were symptomatic. Furthermore, all patients underwent abdominal ultrasound evaluation of the intestinal loop and those with ultrasound signs of suspected IBD were excluded. Patients with a clinical history of suspected IBD (i.e. presence of rectal bleeding or hematochezia) also underwent a complete ileo-colonoscopy. IBD diagnosis was excluded in these when both endoscopy and histology were negative.

Elimination diet and double-blind placebo-controlled (DBPC) challenge

On entering the study, all patients commenced a standard elimination diet, which excluded wheat, cow's milk, eggs, tomato and chocolate. Patients self-reporting food hypersensitivity were also asked to avoid ingestion and/or contact with any food(s) causing symptoms. Food diaries were kept during the elimination diet period to assess dietary intake and adherence to the diet. After four weeks of elimination diet, DBPC challenges were performed, with the reintroduction of a single food at a time. Patients were randomized to receive either the "active food" or the placebo, according to a computer-generated order determined by an observer not involved in the study.

The challenge vehicles were changed over time. Between January 2006 and June 2011, wheat challenge was performed by administering a daily dose 13g of wheat flour, equal to about 20g of bread, or rice flour as placebo, in capsules. A total of 12 capsules daily were given subdivided into three doses, away from meals.

From July 2011 up to the end of the study period, the DBPC challenge was performed with sachets of flour coded A or B containing wheat flour or rice flour, respectively. Sachets A or B were given for 2 consecutive weeks, and then after 1 week of washout patients received the other sachets for another 2 weeks (cross-over design). If needed, the washout period was eventually extended for a maximum of another 2 weeks, until the symptoms induced by the previous challenge had completely resolved, before starting the next challenge. Wheat challenge was performed by administering a daily dose of 80g of flour, which was dissolved and cooked by the patients themselves. Wheat sachets contained 6.5g of gluten. The codes of the sachets and capsules were broken only at the end of the study and the investigators did not know their contents during the study period.

Challenges for other foods in patients with suspected multiple food hypersensitivity were performed in an open fashion.

During the challenge period, the severity of intestinal and extra-intestinal symptoms was recorded: patients completed a 100mm visual analog scale (VAS, with 0 representing no symptoms and 10 intolerable symptoms), which assessed overall symptoms and the specific symptoms they each reported.

The challenges were stopped when clinical reactions occurred for at least two consecutive days [increase in VAS score >30: both for Irritable Bowel Syndrome (IBS)-like symptoms - onset of abdominal discomfort or pain, associated with a change in stool frequency and/or stool appearance - and for extra-intestinal symptoms]. Challenges were considered positive if the same symptoms which had been initially present reappeared after their disappearance on elimination diet and if the VAS score increased by >30 when compared to any eventual increase during the placebo administration.
